# Nitric oxide-based protein modification: formation and site-specificity of protein *S*-nitrosylation

**DOI:** 10.3389/fpls.2013.00137

**Published:** 2013-05-14

**Authors:** Izabella Kovacs, Christian Lindermayr

**Affiliations:** Institute of Biochemical Plant Pathology, Helmholtz Zentrum München, German Research Center for Environmental HealthMunich, Germany

**Keywords:** protein *S*-nitrosylation, nitric oxide, post-translational modification, cysteine residue, redox-modification, site-specificity

## Abstract

Nitric oxide (NO) is a reactive free radical with pleiotropic functions that participates in diverse biological processes in plants, such as germination, root development, stomatal closing, abiotic stress, and defense responses. It acts mainly through redox-based modification of cysteine residue(s) of target proteins, called protein *S*-nitrosylation.In this way NO regulates numerous cellular functions and signaling events in plants. Identification of *S*-nitrosylated substrates and their exact target cysteine residue(s) is very important to reveal the molecular mechanisms and regulatory roles of *S*-nitrosylation. In addition to the necessity of protein–protein interaction for *trans*-nitrosylation and denitrosylation reactions, the cellular redox environment and cysteine thiol micro-environment have been proposed important factors for the specificity of protein *S*-nitrosylation. Several methods have recently been developed for the proteomic identification of target proteins. However, the specificity of NO-based cysteine modification is still less defined. In this review, we discuss formation and specificity of *S*-nitrosylation. Special focus will be on potential *S*-nitrosylation motifs, site-specific proteomic analyses, computational predictions using different algorithms, and on structural analysis of cysteine *S*-nitrosylation.

## NITRIC OXIDE SIGNALING

The free radical nitric oxide (NO) as a unique gaseous second messenger controls fundamental biological functions in animals, plants, and microbes. NO was discovered as an endothelial-derived relaxing factor, that induced vascular relaxation in smooth muscle cells (Ignarro et al., 1987). Later, the identification of an amino acid L-arginine as a substrate to produce NO gas by a family of NO synthases in mammals and the generation of cyclic guanosine monophosphate (cGMP) after the activation of soluble guanylate cyclase (sGC) by NO (Palmer et al., 1988; Alderton et al., 2001; Cary et al., 2006), has led to the identification of a broad-spectrum of functions of NO in the cardiovascular, immune, nervous system, and human pathologies (Pacher et al., 2007). According to the recent knowledge on NO signaling in animals, the mode of action of NO is divided into three mechanisms. (I) The “classical” NO signaling, which is dependent upon sGC and its related enzymes. (II) The “less classical” NO signaling operates through the inhibition of cytochrome *c* oxidase in mitochondria. Both signaling mechanisms (I and II) rely on direct binding of NO to protein metal centers through coordination chemistry. (III) The “non-classical” NO signaling is cGMP independent and related to the NO-mediated post-translational modifications of downstream proteins (Martinez-Ruiz et al., 2011).

With regard to higher plants, numerous studies have also shown the essential role of NO in growth and development (Astier et al., 2012), including seed germination (Bethke et al., 2006; Belenghi et al., 2007), primary and lateral root growth (Correa-Aragunde et al., 2004; Zhao et al., 2007), stomatal closure (Neill et al., 2002), flowering (He et al., 2004), pollen-tube growth regulation (Prado et al., 2004), and fruit ripening and senescence (Leshem et al., 1998; Corpas et al., 2004). Furthermore, NO is a crucial component of plant immune responses (Gaupels et al., 2011; Yu et al., 2012) and various abiotic stresses (Corpas et al., 2011). Despite the extensive studies on NO function in different processes of plants, the whole picture of NO impact on living cells, including production, activity, and metabolism of NO still has yet to be completed. Different mechanisms of NO signaling, according to the animal classification, have also been reported in plants. Ca^2+^ and cGMP are also involved in NO signaling as second messengers in response to biotic (Durner et al., 1998; Klessig et al., 2000; Ma et al., 2009) and abiotic stresses (Martinez-Atienza et al., 2007). However, the most studied mode of action of NO is protein *S*-nitrosylation, the covalent attachment of an NO group to the thiol site of protein cysteine. Protein *S*-nitrosylation, as a reversible post-translational modification affects protein activity (either by activation or inhibition), translocation and protein function. In this review, we discuss the mode of action of NO focusing on the formation and site-specific analysis of *S*-nitrosylation.

## *S*-NITROSYLATION AS A POST-TRANSLATIONAL MODIFICATION

The ability of NO to diffuse across membranes, in addition to its radical nature, leads to the wide range of interactions with biological targets in a concentration and redox-dependent fashion. Investigations of different signaling events have revealed that temporal as well as spatial regulation of NO is required for efficient signal transduction. Protein *S*-nitrosylation has been established as a significant route by which NO transmits its ubiquitous cellular influence (Hess et al., 2005). Furthermore, other major types of modifications of NO have also been reported (Martinez-Ruiz et al., 2011; Astier and Lindermayr, 2012; Toledo and Augusto, 2012), such as metal nitrosylation or tyrosine nitration. The latter one is an irreversible reaction of a nitrating agent with a tyrosine residue of a target protein (Ford, 2010).

Furthermore, to make the signaling function of *S*-nitrosylation even more complex, a recent review in the animal field has summarized a growing number of examples, where the major protein post-translational modifications (e.g., phosphorylation, ubiquitylation, acetylation, sumoylation, palmitoylation) have also been affected and regulated by *S*-nitrosylation *via* signal crosstalks (Hess and Stamler, 2012). Besides *S*-nitrosylation, increasing interest and evidence point at the important role of protein denitrosylation as a feedback mechanism controlling NO signaling. The removal of NO group from proteins might occur through different enzyme systems, of which especially *S*-nitrosoglutathione (GSNO) reductase and thioredoxin play a predominant role (Benhar et al., 2009). Additionally, non-enzymatic denitrosylation has been described, for example, in response to exposure to heat, light, reducing agents, nucleophilic compounds, or by transition metals. As a result of complex functions of *S*-nitrosylation with different levels of regulation, the literature encompasses around 3000 *S*-nitroso proteins (including those identified by exogenous treatment, physiological, or by nitrosylating agents) from animals and plants, which highlight the importance of protein *S*-nitrosylation in a broad-spectrum of cellular processes (Hess and Stamler, 2012).

In recent years, much effort has been made to identify *S*-nitrosylated proteins in plants. A number of candidates have been identified from proteome-wide analyses using NO donors as *S*-nitrosylating agents (**Table 1**). These studies were based on the biotin switch technique (BST), which was the first assay designed to detect *S*-nitrosylated (SNO) proteins from cells and tissues. BST is a three-step method to convert SNO cysteines into biotinylated cysteine residues that easily be detected using streptavidin or a specific antibody (Jaffrey and Snyder, 2001). In the first step, the reduced protein thiols are blocked under denaturing conditions with *S*-thiomethylating agents, such as monomethyl thiosulfonate (MMTS), *N*-ethylmaleimide, or iodo-acetic acid. Following the blocking step, the SNO-bond is specifically reduced to a free thiol with ascorbate. Finally, free thiols are reacted with a thiol-specific reversible biotinylating agent, such as biotin-HPDP. Biotinylated proteins are visualized directly using an avidin antibody. Alternatively, biotinylated proteins are precipitated with immobilized avidin or streptavidin and analyzed by Western blotting for protein-of-interest or by mass spectrometry (MS). After the introduction of BST, increasing numbers of modifications have been reported due to some critical steps in the assay, which may result in false-positive detection of SNO sites. For example, in the first step it is crucial to minimize background biotinylation due to incomplete blocking and to maximize assay sensitivity (Forrester et al., 2009a). However, the most controversial issue is the efficiency and specificity of ascorbate in the second reduction step. Zhang et al. (2005) have reported that ascorbate is a very inefficient reducer of protein-SNOs and high ascorbate concentrations and long incubation times are necessary to achieve a quantitative reaction. Other studies on ascorbate specificity have shown that ascorbate accelerates the rate of the biotinylation reaction and increases the presence of false-positive signals (Huang and Chen, 2006) or can even reduce protein disulfides as in the case of microtubule proteins (Landino et al., 2006). These controversial results could be partially explained by the study of Forrester et al. (2007), who reported that the exposure of samples to indirect sunlight from a laboratory window could reduce the activated disulfide in biotin-HPDP and facilitate biotinylation of MMTS-blocked protein thiol. Ascorbate can also catalyze the Cu-dependent reduction of SNOs to increase the specificity of BST (Wang et al., 2008), whereas it acts as a nucleophile in the absence of added metal ions (Holmes and Williams, 2000). Despite the controversial reports, the BST is the most widely used method of detection of SNO proteins in biological samples. The identified proteins from plant proteome-wide studies have been shown to take part in major cellular activities, notably primary and secondary metabolism, photosynthesis, protein folding and genetic information processing, cellular architecture, and response to biotic and abiotic stresses (Astier et al., 2012). Although the growing number of *S*-nitroso proteins that have been revealed, the characterization of these candidates needs confirmation by candidate-specific approaches in respect of their physiological relevance. To date, around 20 different *S*-nitrosylated proteins have been characterized in details in plants and most of them have been reviewed recently with regard to their functional significance in NO signaling (Astier et al., 2012; Yu et al., 2012).

**Table 1:** Summary of proteomics approaches to identify candidate proteins for *S*-nitrosylation in plants.

Plant species	Tissue/organelle	Treatment	No. of identified candidates	Reference
*Arabidopsis thaliana*	Cell cultures	GSNO-treated protein extracts	63	Lindermayr et al. (2005)
*Arabidopsis thaliana*	Leaves	Plants treated with gaseous NO	52	Lindermayr et al. (2005)
*Kalanchoe pinnata*	Leaves	GSNO-treated protein extracts	19	Abat et al. (2008)
*Arabidopsis thaliana*	Leaf mitochondria	GSNO-treated protein extracts	11	Palmieri et al. (2010)
*Arabidopsis thaliana*	Cell cultures	GSNO-treated protein extracts	27	Maldonado-Alconada et al. (2011)
*Arabidopsis thaliana*	Leaves	*Pseudomonas syringae* avir/vir	119	Maldonado-Alconada et al. (2011)
*Arabidopsis thaliana*	Cell cultures	untreated	53	Fares et al. (2011)
*Solanum tuberosum*	Leaves	GSNO-treated protein extracts	34	Kato et al. (2012)
*Solanum tuberosum*	Tubers	GSNO-treated protein extracts	46	Kato et al. (2012)
*Oryza sativa* (WT)	Leaves	High light	73	Lin et al. (2012)
*Oryza sativa* (nitric oxide\hb excess1 mutant; noe1)	Leaves	High light	100	Lin et al. (2012)
*Pisum sativum*	Leaf peroxisomes	GSNO-treated peroxisomes	6	Ortega-Galisteo et al. (2012)
*Pisum sativum*	Leaf mitochondria	Salt-stressed plants	24	Camejo et al. (2012)

## FORMATION OF *S*-NITROSOTHIOLS

The mechanism of formation of *S*-nitrosothiols *in vivo* is an important factor in understanding the biological actions of NO. The intrinsic biochemistry of NO suggests multiple reaction pathways for *S*-nitrosylation mechanisms based on various *in vitro* studies. Most of these studies have used thiol-containing molecules like cysteine and glutathione for *S*-nitrosylation to yield low molecular weight *S*-nitrosothiols such as *S*-nitrosocysteine and GSNO (Gow et al., 1997; Keszler et al., 2010). They form an integral part of the total cellular nitrosothiol (RSNO) pool and have potential roles as intermediates in transport, storage, and delivery of NO.

NO as a free radical(^·^NO) can lose or gain electrons to become an oxidized nitrosonium cation (NO^+^) or a reduced nitroxyl anion (NO^-^), each with a different oxidation state of the nitrogen atom (+2, +3, and +1, respectively; Arnelle and Stamler, 1995). Furthermore, NO can be oxidized in an aerobic, biological milieu up to its +5 oxidation state yielding non-reactive nitrate anions(${NO}_{3}^{-}$). The generation and the presence of different redox status of NO multiply the possibilities to produce *S*-nitrosylated proteins in numerous conditions.

**FIGURE 1:**
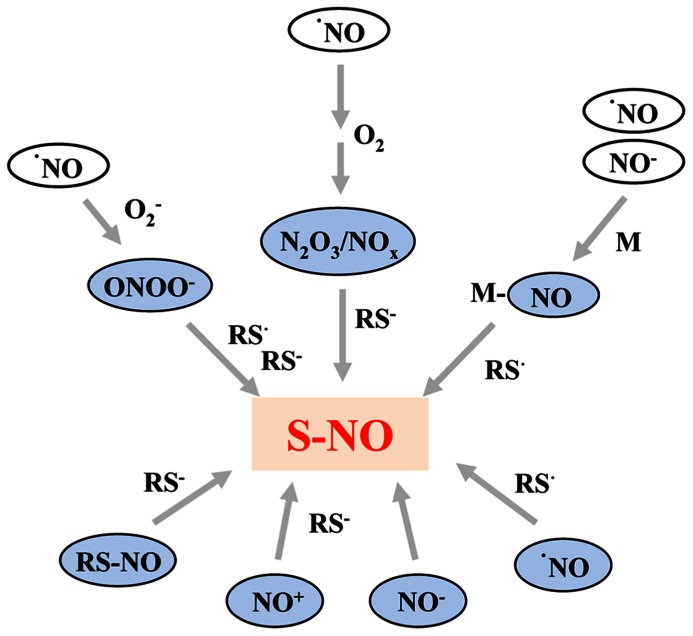
**Summary of the most important pathways resulting in formation of *S*-nitrosothiols**. NO as free radical (^·^NO) reacts primarily with superoxide ($O_{2}^{-}$), oxygen (O_2_), and redox metals (M) resulting in formation of *S*-nitrosylating agents, such as peroxynitrite (ONOO^-^), NO oxides (N_2_O_3_/NO_x_), or metal–NO complexes (M–NO). The latter can also be formed by the reaction of nitroxyl anions with redox metals. Furthermore, NO radicals can react directly with thiyl radicals (RS^·^). Moreover, the oxidized nitrosonium cation (NO^+^) and the reduced nitroxyl anion (NO^-^) can act as *S*-nitrosylating agents. However, the direct reaction between thiols and NO^-^ is dependent on the energy state of NO^-^ and occurs only when NO^-^ is present in the high energy singlet state. The thiol groups and thiyl radicals can either be part of a protein or a low molecular weight compound such as glutathione.

^·^NO is a poor oxidant and also a poor reducing agent under physiological conditions, therefore NO-dependent amino acid oxidation mostly occurs *via* secondary reactions after the oxidation of NO to nitrogen dioxide (NO_2_),dinitrogen trioxide (N_2_O_3_), or peroxynitrite (ONOO^-^) in the presence of oxygen or reactive oxygen species (Broniowska and Hogg, 2012). There are four major mechanisms of *S*-nitrosylation that have the potential to occur in biological systems: (1) an oxidative pathway with NO in a higher oxidation status, (2) a radical-mediated pathway with ^·^NO and thiyl (RS^·^) radicals, (3) metal-catalyzed RSNO formation in the presence of transition metals, and (4) *trans*-nitrosylation. The different reactions are summarized in **Figure 1**.

### THE OXIDATIVE PATHWAY OF *S*-NITROSYLATION

In this pathway, *S*-nitrosylation is modulated by higher oxides of NO, such as NO^+^ and N_2_O_3_. RSNO can be formed by the direct addition of NO^+^ to a thiol at neutral pH, but the major limitation of this mechanism is that nitrosonium is unstable in water, immediately hydrolyzing to nitrite. Therefore, the thiol must be in the immediate vicinity of the source of NO^+^ (Kettenhofen et al., 2007). N_2_O_3_ is generally considered as a nitrosylating agent that can directly mediate thiol nitrosylation (Wink et al., 1994; Goldstein et al., 1996) attacking the reduced (nucleophilic) thiolate anion (RS^-^) to yield the *S*-nitrosylated product and nitrite. To generate N_2_O_3_, ^·^NO must be oxidized to NO_2_ by oxygen, which combines with ^·^NO to form N_2_O_3_. This aerobic formation of N_2_O_3_ depends on the concentration of available^·^NO and O_2_, because this reaction is of second order in ^·^NO and of first order in O_2_ (Goldstein et al., 1996), and consequently very slow at biological concentrations of ^·^NO. It has been suggested that hydrophobic areas in membranes and proteins could increase the local concentration of both ^·^NO and O_2_ to accelerate this reaction (Nedospasov et al., 2000). However, Keszler et al. (2010) have recently reported contradictory results with the conclusion that protein hydrophobic environments do not enhance *S*-nitrosylation.

N_2_O_3_ could also be formed from the condensation of nitrous acid (HNO_2_; Guikema et al., 2005). The p*K*_ a_ of HNO_2_ is approximately 3.4, at higher pH values HNO_2_ dissociates into nitrite (${NO}_{2}^{-}$). Since N_2_O_3_ formation from HNO_2_ can occur only at low acidic pH, the physiological relevance to produce RSNO on this way at neutral pH is still questionable. However, the apoplast of plants is acidic and might be mediating pH-dependent synthesis of N_2_O_3_, and it was indeed reported that NO could be produced from nitrite in an acidified apoplast (Bethke et al., 2004).

### RADICAL-MEDIATED PATHWAY OF *S*-NITROSYLATION

Radicals play an important role in mediating cellular signaling processes during stress responses. The direct combination of ^·^NO and thiol results in putative intermediate radicals (RSN^·^OH), which in the presence of an electron acceptor get oxidized to *S*-nitrosothiols and superoxide (Gow et al., 1997). However, the major radical–radical combination reaction is the addition of ^·^NO to a thiyl radical (RS^·^) to form *S*-nitrosothiols. Rate measurements have demonstrated that such a radical combination reaction is extremely fast (Madej et al., 2008). One-electron oxidation of ^·^NO yields ^·^NO_2_, which can oxidize thiols to thiyl radicals (Jourd’heuil et al., 2003; Keszler et al., 2010). In addition, ONOO^-^, the product of ^·^NO and superoxide ($O_{2}^{-}$), can form *S*-nitrosothiols either directly with thiolate anion (RS^-^; van der Vliet et al., 1998) or through thiyl radicals (Goldstein et al., 1996; Keszler et al., 2010). So, any mechanism or cellular processes that could enhance RS^·^ formation, such as increased superoxide formation or the action of peroxidases, has the potential to generate *S*-nitrosothiols, too. However, generation of one-electron oxidants in general requires oxygen, although some data indicate that under certain conditions cellular RSNO formation can occur without O_2_ (Bosworth et al., 2009). It was recently shown that endogenous *S*-nitrosylation during anaerobic respiration is also controlled by the transcription factor OxyR, previously thought to operate only under aerobic conditions (Seth et al., 2012).

### METAL-CATALYZED SNO FORMATION

Redox-active metal ions can catalyze many of the above mentioned reactions of *S*-nitrosothiol formation. The main sites of action of NO are heme groups and other metal groups like those in sGC, cytochrome *c* oxidase, or hemoglobins (Brandish et al., 1998; Sarti et al., 2000). Proteins with iron-containing prosthetic groups show the fastest reactions with NO. Iron and NO can take part in reversible electron transfer processes depending on the redox environment. Ferric irons (Fe^3^^+^) can accept electrons from radical ^·^NO resulting in the formation of ferrous (Fe^2^^+^) and NO^+^ ions, whereas Fe^2^^+^ can donate electrons to radical ^·^NO to form Fe^3^^+^ and NO^-^ (Graziano and Lamattina, 2005). There is evidence that copper and iron ions are able to generate *S*-nitrosothiols *via* one-electron oxidation of thiols to thiyl radicals or by the formation of NO/metal complexes (Vanin et al., 1997; Stubauer et al., 1999). Fe^2^^+^, NO, and low molecular weight thiols can form metal containing *S*-nitrosothiols *in vivo* called dinitrosyl iron complexes (Mulsch et al., 1993; Shumaev et al., 2008). These complexes are considered endogenous NO carriers like low molecular weight nitrosothiols. They have been shown to transfer NO to the metal-centers of metalloproteins and/or can donate NO^+^ equivalents to thiol groups to form RSNO (Bosworth et al., 2009). It was recently shown that increased NO levels in plants elevate the levels of nitrosyl–iron complexes (Simontacchi et al., 2012).

### *TRANS*-NITROSYLATION

*Trans*-nitrosylation is presumably the most important reaction of an *S*-nitrosothiol inside a cell (Arnelle and Stamler, 1995). In addition to direct modification of thiol groups by NO equivalents, both *S*-nitrosocysteine and *S*-nitrosylated proteins can directly transfer their nitrosyl moiety to acceptor cysteine thiols (Cys-to-Cys transfer). This reversible reaction involves the nucleophilic attack of a thiolate anion (RS^-^) of the acceptor protein on the nitroso nitrogen of the donor molecule. There is evidence that not all of the protein thiols are modified by *trans*-nitrosylation, and some thiols in an individual protein are preferentially *S*-nitrosylated. Nitrosylases can enzymatically mediate *trans*-nitrosylation, but examples of *trans*-nitrosylation catalyzed by metalloproteins (metal-to-Cys transfer) also exist (Anand and Stamler, 2012).

*S*-nitrosoglutathione is the major physiological NO donor among the low molecular weight *S*-nitrosothiols, and it is known for its ability to mediate *trans*-nitrosylation (Dahm et al., 2006). This is the reason why GSNO is the most commonly used NO donor in proteome-wide studies in animals and plants. Furthermore, the emerging role of *trans*-nitrosylation between cellular proteins has been revealed as an important mechanism in cell signaling pathways (Nakamura and Lipton, 2013).

## DETECTION AND IDENTIFICATION OF *S*-NITROSYLATED PROTEINS

Proteomic analyses of *S*-nitrosothiols generated *in vivo* are usually a challenge due to the low level, dynamic, and unstable features of *S*-nitrosylation. Nitrosothiols undergo photolytic degradation (Singh et al., 1996) and are reduced by cytosolic reducing agents (ascorbate, glutathione) or by metals (Smith and Dasgupta, 2000) in biological system. This can explain that most of the published studies on *S*-nitrosylated proteins have relied on exogenous treatments with NO or NO donors, which increase the total intracellular RSNO pool to intensify the effect of NO. The development of new techniques for the enrichment and identification of *S*-nitrosylated proteins and mapping of sites of *S*-nitrosylation (SNO-sites) have led to an increasing number of results in this field over the last 10 years.

Various assays with goal-specific modifications have been developed for the characterization, identification, and quantification of *S*-nitrosylated proteins (Torta et al., 2008; Foster, 2012; Liu et al., 2012). Most of the work has used indirect methods, measuring free NO levels after cleavage of SNO bonds or replacing the original nitrosothiols with another detectable tag (BST). The alternative direct detections of NO-modified thiols by MS or X-ray crystallography have been predominantly applied to the characterization of isolated proteins.

### IDENTIFICATION OF SNO SITES

The most commonly used indirect method to detect *S*-nitrosylated proteins is a BST mentioned above. For a large-scale proteomics analysis, Gross and colleagues have improved the BST technique now called SNO site identification (SNOSID; Hao et al., 2006). In this approach, the proteins are digested with trypsin after labeling of nitrosylated groups with biotin, and the resulting peptides are affinity-purified. The purified peptides are then eluted and analyzed by liquid chromatography–tandem MS (LC–MS/MS), allowing high-throughput identification of the modified cysteines. In another study, the SNOSID has been modified to improve the LC/MS performance to optimize a detergent-free protocol by using urea as a denaturant in the first step of BST from HeLa cells in the presence or absence of GSNO (Han and Chen, 2008). Greco et al. (2006) have reported that using formic acid instead of a reducing agent to elute biotinylated peptides from avidin beads increases the specificity of the detected SNO sites, because in this way the biotin-HPDP adduct is still present on the eluted peptides.

The SNO-resin assisted capture methodology substitutes a thiol-active resin (e.g., thiopropyl sepharose) for biotin-HPDP (Forrester et al., 2009b). This method simplifies SNOSID such that the captured proteins are proteolyzed on resin, and SNO-site-containing peptides are eluted with reductant and identified by LC–MS/MS. The elimination of biotin removal and avidin-based enrichment steps appears to improve the sensitivity for detection of high-mass SNO-proteins.

Another way of modification of BST is the His-tag switch method, where the SNO-bond is specifically replaced by a His-tag containing peptide (Camerini et al., 2007). The advantage of this new labeling peptide is that it binds irreversibly to the reduced cysteines, maintaining the proteins label through all the purification steps. This approach was applied to the analysis of neuronal cytosolic proteins from rat cerebral cortex and 28 *S*-nitrosylated sites were identified by MS.

To circumvent the ascorbate reduction step in the BST, phenylmercury compounds were alternatively used to react directly with *S*-nitrosocysteine forming a relative stable thiol-mercury bond (Doulias et al., 2010). In this study, an organomercury resin was synthesized for solid-phase capturing of *S*-nitrosylated proteins/peptides. A different approach used a newly designed phenylmercury-polyethylene glycol-biotin compound for biotin-avidin affinity purification. In addition to the mercury-based SNO reaction, Faccenda et al. (2010) have described a gold nanoparticle that can be used to directly enrich *S*-nitrosylated proteins. It was demonstrated that thiols and thioethers have a higher affinity for gold than other functional groups in proteins. Thus, these particles may have the potential to discriminate the disulfide bond and other cysteine modifications from the *S*-nitrosylated ones.

### SNO-SITE QUANTIFICATION

Understanding the role of NO modifications under various physiological and pathological conditions requires quantification of the dynamic changes of protein *S*-nitrosylation. Relative levels of SNO-proteins can be determined using Western blotting and densitometry or by fluorescence-based quantification using either thiol-reactive fluorophores (Kettenhofen et al., 2008; Tello et al., 2009) or fluorescent secondary antibodies (Foster et al., 2009). The fluorescence labeling of the modified thiols by two different fluorophores (Cy3 and Cy5) from control and treated samples in combination with a 2D difference gel electrophoresis technique allows the simultaneous identification of a protein from a single spot and the quantification of the relative level of thiol modification (Kettenhofen et al., 2007). Further modification for specifically labeling, detecting, and quantifying protein *S*-nitrosylation is reported using a fluorescence saturation labeling technique and unique concepts for measuring changes in *S*-nitrosylation status relative to protein abundance are introduced (Wiktorowicz et al., 2011).

Mass spectrometry has also been employed for relative quantification of SNO-proteins. The *S*-nitrosothiol capture method adapts the isotope-coded affinity tag technique (Gygi et al., 1999), which utilizes light and heavy isotope-labeled (12C and 13C, respectively) thiol biotinylating agents in conjunction with LC–MS/MS to identify and quantify expression level differences of SNO-sites between two conditions (Paige et al., 2008; Fares et al., 2011). Huang and Chen (2010) reported a combination of isotope-based technique and irreversible biotinylation procedure using a biotin-maleimide tag instead of biotin-HPDP for a quantitative proteomic approach of *S*-nitrosylation. Alternatively, stable isotope labeling of amino acids in cell culture, which employs light and heavy isotope-labeled Arg and/or Lys for relative quantification of tryptic peptides, has been combined with detergent-free BST and LC–MS/MS for endogenous SNO quantifications (Zhou et al., 2010; Torta and Bachi, 2012). The major advantage of this labeling method is that samples can be mixed before the blocking and purification steps thus reducing the errors deriving from sample preparations and providing a more accurate quantification ratio.

## STRUCTURAL FEATURES OF CYSTEINE *S*-NITROSYLATION SITES

Most proteins possess cysteine residues, but the affinity of this amino acid residue to NO can be very different. Despite of proteome-wide studies and *S*-nitrosylation motif screens, up to now, there is no general rule which could explain which cysteine would be amenable to nitrosylation. In the beginning, the linear sequence of *S*-nitrosylated proteins was analyzed searching for consensus motifs. The analysis of NO transfer in hemoglobin provided the basis for an acid–base motif for protein *S*-nitrosylation and denitrosylation (Stamler et al., 1997). The acid–base motif is comprised of flanking acidic (D, E) and basic (R, H, K) residues embracing the reactive thiol cysteine sites ([KRHDE]-C-[DE]). These can suppress or favor, respectively, the formation of nucleophilic thiolate (RS^-^) through electrostatic interactions. This motif has been shown to be predictive in a number (but not all) of cases and the general feature of acid–base motifs is still object of intense discussion since then. Moreover, a GSNO binding motif has been described ([HKR]-C-[hydrophobic]X[DE]) (Hess et al., 2005). In *Arabidopsis* *S*-adenosylmethionine synthetase 1 such a GSNO binding motif for *trans*-*S*-nitrosylation is present, embracing C_114_. *S*-nitrosylation of this cysteine residue is responsible for inhibition of the activity of *S*-adenosylmethionine synthetase 1 (Lindermayr et al., 2006). Another factor suggested to play a role in *S*-nitrosylation is a low p*K*_ a_ of cysteine, for example in the case of metalloproteins, where transition metal coordination decreases thiol p*K*_ a_ (Hess et al., 2005). It has been shown that the interaction between Cys thiols and aromatic side chains in its vicinity promotes formation of a thiolate anion, which enhances the possibility of NO modification (Britto et al., 2002). *S*-nitrosylated cysteines were also found in hydrophobic pockets of proteins (Greco et al., 2006), which can sequester or stabilize radicals to form *S*-nitrosylating species (Nedospasov et al., 2000). With the development of different methods and proteomic approaches for detection and identification of *S*-nitrosylated sites from complex biological mixtures an increasing number of identified SNO sites have allowed to analyze the precise environments of the modified cysteines. A proteomic approach using selective peptide capturing from human vascular smooth muscle cells revealed 18 proteins with *S*-nitrosylated cysteines and the presence of acid–base motifs, as well as hydrophobic motifs surrounding the identified cysteine residues (Greco et al., 2006). In contrast, using the SNOSID method to identify 68 SNO-Cys sites from rat cerebellum protein samples, Hao et al. (2006) failed to find any evidence for a linear Cys-flanking motif with the help of a machine learning approach. They suggested that key determinants of NO-targeting are likely to be encoded in the 3D cysteine environment. Searches of *Arabidopsis* protein databases with a degenerate SNO motif proposed by Stamler [GSTCYNQ]-[KRHDE]-C-[DE] have yielded a few hundred hits which include proteins related to cell signaling, transport, cell cycle, and metabolism processes (Wilson et al., 2008). However, no protein of this list has so far been identified to be *S*-nitrosylated *in vivo*. A novel proteomic approach using site-specific high-throughput identification of protein *S*-nitrosylation from breast cancer cells revealed a consensus I-C hydrophobic motif center flanked by acidic (D/E) and basic (R/K) residues (Liu et al., 2010). This result provided direct support for the presence of the acid–base motif and the importance of a hydrophobic environment in the vicinity of *S*-nitrosylated cysteine (Hess et al., 2001). Marino and Gladyshev (2010) have used a bioinformatic approach to analyze general features of *S*-nitrosylation. A data set of 55 non-redundant proteins containing 70 SNO-Cys sites was created manually based on *in vitro* studies available from literature reports. They included only proteins with established crystal or NMR structures, or proteins that could be modeled by standard homology modeling approaches. They established that the proximal acid–base motif, cysteine p*K*_a_, sulfur atom exposure, and cysteine hydrophobicity in the vicinity of the modified cysteine did not define the specificity of *S*-nitrosylation. Instead, they proposed a revised acid–base motif, which was located more distantly with regard to the Cys (8 Å) and with its charged groups exposed (solvent accessible). Charged residues have a strong influence on the electrostatic potential distribution of proteins, which is a crucial feature for protein–protein interactions. They hypothesize that the modified acid–base motif plays a role in protein–protein interactions resulting in *trans*-nitrosylation of target proteins rather than in the direct activation of cysteine to form thiolate anions for further *S*-nitrosylation (Marino and Gladyshev, 2010). Doulias et al. (2010) reported an endogenous *S*-nitrosoproteome study from WT mouse liver with 328 modified Cys sites in 192 proteins suggesting multiple mechanisms for selective site-directed *S*-nitrosylation. Structural analysis of *S*-nitrosylated cysteines revealed that SNO sites were equally distributed in hydrophobic and hydrophilic areas of proteins with an average predicted p*K*_a_ of 10.01. Furthermore, 70% of the *S*-nitrosylated cysteine residues were surrounded by negatively or positively charged amino acids within a distance of 6 Å. Based on the presence of modified cysteine residues in highly exposed areas of proteins and in proximity to charged amino acids they suggest a protein or small molecule (like GSNO) *trans*-nitrosylation assisted mechanism (Doulias et al., 2010). Moreover, 13 modified cysteines were coordinated with metals and 15 metalloproteins were endogenously modified supporting metal-catalyzed *S*-nitrosylation mechanisms, too. Proteomics investigation of endogenous *S*-nitrosylation in *Arabidopsis* cell suspensions yielded 53 SNOSIDs (Fares et al., 2011). Structural studies of *S*-nitrosylated cysteines and their vicinities have shown no clear over-representation of acid–base motifs, only three apolar residues (A-Ala, G-Gly, I-Ile) were found to be significantly enriched in the flanking regions of the modified cysteines.

## COMPUTATIONAL PREDICTION OF *S*-NITROSYLATION SITES

However, although much effort was paid to find consensus structural features to describe the specificity of *S*-nitrosylation based on a large number of datasets from a different proteomic studies, the prediction of *S*-nitrosylation sites in proteins still remains a great challenge. In the case of other post-translational modifications, computational approaches have been shown to be able to rapidly generate helpful information to stimulate further experimental verification. Xue et al. (2010) have first developed the software called GPS-SNO 1.0 for *S*-nitrosylation site prediction. They have improved their previously developed algorithm for the prediction of kinase-specific phosphorylation sites and released the GPS 3.0 algorithm (group-based prediction system) for GPS-SNO. The training set for the new algorithm was obtained from the literature and from public databases with 504 experimentally verified *S*-nitrosylation sites in 327 unique proteins. The prediction performance of GPS 3.0 yielded an accuracy of 75.80%, a sensitivity of 53.57% and a specificity of 80.14% under low threshold condition. GPS-SNO 1.0 was applied on a test set of 485 potentially *S*-nitrosylated substrates collected from PubMed. The SNO site prediction resulted in at least one potential *S*-nitrosylation site per protein of 74% of the test proteins, which could be a starting point for further experimental verifications. The online service and local packages of GPS-SNO 1.0 are freely available at http://sno.biocuckoo.org/.

Another systematic informatics investigation on the *S*-nitrosylation substrate specificity was reported by Lee et al. (2011). The study was based on 586 *S*-nitrosylation sites identified experimentally from mouse endothelial cells (Chen et al., 2010). Site-specific characterization including structural factors such as the flanking amino acids composition, the accessible surface area and physicochemical properties was needed to distinguish the *S*-nitrosylation sites from non-*S*-nitrosylation sites. Due to the difficulty to obtain the conserved motifs by conventional motif analysis, maximal dependence decomposition (MDD) was applied to obtain statistically significant conserved motifs to cluster all sequences of *S*-nitrosylation sites into 11 subgroups. Support vector machine (SVM) was applied to generate predictive model for each MDD-clustered motif. According to fivefold cross-validation, the MDD-clustered SVMs could achieve an accuracy of 0.902, and provided a promising performance on an independent test set (experimental *S*-nitrosylation data from GPS-SNO). The models obtained with the MDD clustering method were applied to implement a novel web-based tool, named SNOSite for identifying cysteine *S*-nitrosylation. SNOSite can be accessed *via* a web interface, and is freely available at http://csb.cse.yzu.edu.tw/SNOSite/. Databases of cysteine *S*-nitrosylation have also been established to collect experimentally determined SNO sites. The dbSNO database was created to integrate all available datasets and to provide their structural analysis (Lee et al., 2012). Up to April 15, 2012, the dbSNO has manually accumulated >3000 experimentally verified *S*-nitrosylated peptides from 219 research articles using a text mining approach. The dbSNO database provides structural and functional analyses, including the motifs of substrate sites, solvent accessibility, protein secondary and tertiary structures, protein domains, and gene ontology. The dbSNO is freely accessible *via* http://dbSNO.mbc.nctu.edu.tw. Another database named SNObase released nearly at the same time has collected *S*-nitrosylation targets extracted from the literature up to June 1, 2012 (Zhang et al., 2012). SNObase contains 2561 instances, and provides information about *S*-nitrosylation targets, sites, biological model, related diseases, trends of *S*-nitrosylation level, and effects of *S*-nitrosylation on protein function. *S*-nitrosylation targets from plants represent 6.4% of the total database, whilst the majority are human (41.5%), and mouse (32.6%). SNObase is freely available at http://www.nitrosation.org.

Recently another predictor, called iSNO-PseAAC (pseudo amino acid composition), has been published for identifying the SNO sites in proteins by incorporating the position-specific amino acid propensity into the general form of PseAAC (Xu et al., 2013). The benchmark dataset was derived from the dbSNO database. To reduce the redundancy, 438 proteins were selected (with 731 experimentally verified SNO sites) in which none had ≥40% pairwisesequence identity to any other. The overall cross-validation success rate in identifying nitrosylated proteins on an independent dataset was over 90%. A web-server for iSNO-PseAAC was established at http://app.aporc.org/iSNO-PseAAC/.

We have chosen 10 known *S*-nitrosylated proteins from *Arabidopsis thaliana* to compare the prediction efficiency of the available SNO site prediction programs GPS-SNO, SNOSite, and iSNO-PseAAC (**Table 2**). The selected *Arabidopsis* proteins have been analyzed in details for cysteine modifications and *S*-nitrosylation sites have been confirmed by MS. **Table 2** shows that all of the computational programs have predicted SNO sites to the selected proteins with different efficiency. The overall success rate (how many proteins were predicted containing at least one SNO site) was 100% using SNOsite and 90% in the case of both GPS-SNO and iSNO-PseAAC programs. However, the prediction specificities were quite different using the three programs. The SNOSite has predicted a possibility to be modified by NO almost to all of the available cysteines, whereas the GPS-SNO predicted fewer SNO sites with the highest matching to the verified sites. The prediction by iSNO-PseAAC has resulted in the lowest specificity to the identified SNO sites. The GPS-SNO program seems to be the most reliable one for predicting putative *S*-nitrosylation sites providing a good base for future experimental confirmations of proteins with unknown positions of modification.

**Table 2:** Computational prediction of *S*-nitrosylation sites from 10 experimentally identified *S*-nitrosylated proteins from *Arabidopsis thaliana* using GPS-SNO, SNOSite, and iSNO-PseAAC programs.

Proteins	Identified Cys-NO sites by MS	Cys-NO site prediction by GPS-SNO	Cys-NO site prediction by SNOSite	Cys-NO site prediction by iSNO-PseAAC	Total number of Cys	Reference
Methionine adenosyltransferase 1	**C**_114_	**C**_114_	C_20_, C_31_, C_42_, C_73_, C_90_, **C**_114_, C_161_	C_161_	8	Lindermayr et al. (2006)
Metacaspase 9	**C**_147_	C_17_, **C**_147_	C_17_, C_29_, C_117_, **C**_147_, C_309_	C_17_, C_29_	7	Belenghi et al. (2007)
Peroxiredoxin II E	**C**_121_	**C**_121_	**C**_121_, C_146_	**C**_121_, C_146_	2	Romero-Puertas et al. (2007)
NPR1	**C**_156_	**C**_156_, C_385_	C_82_, C_150_, C_155_, **C**_156_, C_160_, C_212_, C_223_, C_297_, C_306_, C_378_, C_385_, C_394_, C_457_, C_511_, C_529_	C_212_, C_306_	17	Tada et al. (2008)
GAPDH	**C**_156_, **C**_160_	**C**_156_, **C**_160_	**C**_156_, **C**_160_	–	2	Holtgrefe et al. (2008)
SABP3	**C**_280_	C_34_, C_173_, **C**_280_	C_34_, C_167_, C_173_, C_230_, C_257_, C_277_, **C**_280_	C_230_, C_257_	7	Wang et al. (2009)
Transcription factor-TGA1	**C**_172_, **C**_260_, **C**_266_, **C**_287_	**C**_172_	**C**_172_, **C**_260_, **C**_266_, **C**_287_	**C**_172_	4	Lindermayr et al. (2010)
NADPH oxidase	**C**_890_	–	C_208_, C_410_, C_412_, C_433_, C_480_, C_651_, C_695_, C_825_, **C**_890_	C_208_, C_387_, C_433_, C_480_, C_695_	10	Yun et al. (2011)
TIR1	**C**_140_	C_516_, C_551_, *C_140_	C_34_, C_44_, C_53_, C_121_, **C**_140_, C_155_, C_193_, C_210_, C_264_, C_269_, C_288_, C_311_, C_337_, C_371_, C_405_, C_480_, C_491_, C_516_, C_523_, C_551_	C_34_, C_53_, C_121_, **C**_140_, C_155_, C_210_, C_269_, C_288_, C_311_, C_405_, C_480_, C_491_	23	Terrile et al. (2012)
cALD2	**C**_173_	C_68_, C_326_, *C_130_, *C_197_	C_68_, **C**_173_, C_197_, C_208_, C_326_	C_326_	6	van der Linde et al. (2011)

*C in bold: matched cysteines*.

*Prediction with low threshold (the recommended medium threshold was used in all of the other cases of GPS-SNO prediction). NPR1, non-expresser of pathogenesis related genes 1; GAPDH, glyceraldehyde 3-phosphate dehydrogenase; SABP3, salicylic acid binding protein 3; TGA1, TGACG motif binding factor; TIR1, transport inhibitor response 1; cALD2, cytosolic fructose 1,6-bisphosphate aldolase.

## CONCLUSION

Protein *S*-nitrosylation, as a redox-based post-translational modification plays an essential role in regulating a number of fundamental and pathological processes in animals and plants. However, many questions still remain in terms of NO production and exact signaling routes, through which NO influences a broad-spectrum of inter- and intracellular signaling in plants. Rapid progress has been made in development and application of proteomic approaches to identify and analyze *S*-nitrosylation in the plant field. Future research needs to focus on characterization of the biological functions of *S*-nitrosylated proteins, e.g., the impact of *S*-nitrosylation on enzyme activities, protein translocation, and protein interaction networks. Moreover, individual *S*-nitrosylation site have to be characterized using a combination of computational predictions and experimental verifications to understand the molecular mechanism of *S*-nitrosylation.

## Conflict of Interest Statement

The authors declare that the research was conducted in the absence of any commercial or financial relationships that could be construed as a potential conflict of interest.
