# Erratum: Nitric oxide-based protein modification: formation and site-specificity of protein S-nitrosylation

**DOI:** 10.3389/fpls.2013.00229

**Published:** 2013-07-04

**Authors:** Izabella Kovacs, Christian Lindermayr

**Affiliations:** Group Redox-Proteomics, Helmholtz Zentrum München - German Research Center for Environmental Health, Institute of Biochemical Plant PathologyMunich, Germany

After our article was published online, we recognized that the summary of proteomics approaches to identify candidate proteins for S-nitrosylation in plants (Table 1) was incomplete. We have included here the following missing references: Romero-Puertas et al., 2008; Abat and Deswal, 2009; Tanou et al., 2009; Tanou et al., 2012.

**Table 1:** **Summary of proteomics approaches to identify candidate proteins for S-nitrosylation in plants**.

**Plant species**	**Tissue/Organelle**	**Treatment**	**No. of identified candidates**	**Citation**
*Arabidopsis thaliana*	Cell cultures	GSNO-treated protein extracts	63	Lindermayr et al., 2005
*Arabidopsis thaliana*	Leaves	Plants treated with gaseous NO	52	Lindermayr et al., 2005
*Arabidopsis thaliana*	Leaves	*Pseudomonas syringae* avirB, HR reaction	16	Romero-Puertas et al., 2008
*Kalanchoe pinnata*	Leaves	GSNO-treated protein extracts	19	Abat et al., 2008
*Brassica juncea*	Seedlings	GSNO-treated protein extracts	20	Abat and Deswal, 2009
*Brassica juncea*	Seedlings	Low temperature	10	Abat and Deswal, 2009
*Citrus aurantium* L.	Leaves	Salt stress	49	Tanou et al., 2009
*Arabidopsis thaliana*	Leaf mitochondria	GSNO-treated protein extracts	11	Palmieri et al., 2010
*Arabidopsis thaliana*	Cell cultures	GSNO-treated protein extracts	27	Maldonado-Alconada et al., 2011
*Arabidopsis thaliana*	Leaves	*Pseudomonas syringae* avir/vir	119	Maldonado-Alconada et al., 2011
*Arabidopsis thaliana*	Cell cultures	Untreated	53	Fares et al., 2011
*Solanum tuberosum*	Leaves	GSNO-treated protein extracts	34	Kato et al., 2012
*Solanum tuberosum*	Tubers	GSNO-treated protein extracts	46	Kato et al., 2012
*Oryza sativa* (WT)	Leaves	High light	73	Lin et al., 2012
*Oryza sativa* (nitric oxide excess1 mutant; noe1)	Leaves	High light	100	Lin et al., 2012
*Pisum sativum*	Leaf peroxisomes	GSNO-treated peroxisomes	6	Ortega-Galisteo et al., 2012
*Pisum sativum*	Leaf mitochondria	Salt-stressed plants	24	Camejo et al., 2012
*Citrus aurantium* L.	Leaves	GSNO-treated protein extracts	82	Tanou et al., 2012
*Citrus aurantium* L.	Roots	GSNO-treated protein extracts	64	Tanou et al., 2012
